# Distinct changes in plasma protein profiles of cancer patients receiving immune checkpoint inhibitors versus chemotherapy: implications for thrombosis risk and adverse outcomes

**DOI:** 10.1016/j.rpth.2026.106929

**Published:** 2026-08-27

**Authors:** Eleonora Camilleri, Nikola Vladic, Cornelia Englisch, Daniel Steiner, Matthias Preusser, Anna S. Berghoff, Thorsten Fuereder, Julia M. Berger, L. Renee Ruhaak, Suzanne C. Cannegieter, Bart J.M. van Vlijmen, Frederikus A. Klok, Cihan Ay

**Affiliations:** 1 Department of Clinical Epidemiology, Leiden University Medical Center, Leiden, The Netherlands; 2 Division of Hematology and Hemostaseology, Department of Medicine I, Medical University of Vienna, Vienna, Austria; 3 Division of Oncology, Department of Medicine I, Medical University of Vienna, Vienna, Austria; 4 Christian Doppler Laboratory for Personalized Immunotherapy, Department of Medicine I, Medical University of Vienna, Vienna, Austria; 5 Department of Clinical Chemistry and Laboratory Medicine, Leiden University Medical Center, Leiden, The Netherlands; 6 Division of Thrombosis and Hemostasis, Department of Medicine, Leiden University Medical Center, Leiden, The Netherlands; 7 Einthoven Laboratory for Vascular and Regenerative Medicine, Leiden University Medical Center, Leiden, The Netherlands

**Keywords:** blood coagulation, cancer, chemotherapy, immune checkpoint inhibitors, venous thrombosis

## Abstract

**Background:**

Chemotherapy and immune checkpoint inhibitors (ICIs) are distinct anticancer treatments. We studied their impact on plasma protein profiles in cancer patients, potentially providing insight into treatment-specific prothrombotic mechanisms.

**Objectives:**

To compare baseline and longitudinal changes in protein profiles of patients with non–small cell lung cancer or head and neck cancer receiving ICI or chemotherapy.

**Methods:**

We analyzed a matched cohort of 30 treatment-naïve patients from the Vienna Cancer and Thrombosis and Bleeding (CAT-BLED) study (10 non–small cell lung cancer and 5 head and neck cancer pairs). Plasma was collected before treatment, after 3 weeks, and at 3 months. We used quantitative protein mass spectrometry to measure 159 protein levels. Longitudinal changes were evaluated using linear mixed models with multiple testing correction.

**Results:**

Baseline protein profiles clustered by treatment assignment. Patients starting ICIs showed higher immune-related protein levels (eg, CD5 antigen-like, immunoglobulin mu chain C) and lower coagulation protein levels (factor[F]X, prothrombin) than those initiating chemotherapy. Two proteins changed during ICI, while 34 proteins (21%)changed during chemotherapy, with none and 12 (7%) remaining after multiple testing correction, respectively. At 3 months, chemotherapy was associated with increased levels of activated factor XIII (FXIIIa) (mean difference: 0.30; 95% confidence interval [CI], 0.06-0.54) and B (0.18; 95% CI, 0.03-0.34), fibulin-1 (0.25; 95% CI, 0.10-0.43), metalloproteinase inhibitor 2 (0.16; 95% CI, 0.02-0.31), and sex hormone-binding globulin (0.71; 95% CI, 0.23-1.14) and decreased serotransferrin (−0.10; 95% CI, −0.18 to −0.01) and cartilage acid protein 1 (−0.21; 95% CI, −0.33 to −0.09).

**Conclusion:**

In this pilot, chemotherapy and ICI were associated with treatment-specific changes in protein profiles, suggesting distinct pathophysiological processes driving thrombosis and adverse outcomes during treatment.

## Essentials

• Chemotherapy and immune checkpoint inhibitors (ICIs) may have distinct prothrombotic mechanisms.
• In 30 cancer patients, 159 plasma proteins were measured longitudinally during treatment.
• Chemotherapy was associated with broader plasma protein changes than ICIs.
• This pilot suggests distinct pathophysiological processes driving thrombosis and adverse outcomes.

## 1 Introduction

Chemotherapy and immune checkpoint inhibitors (ICI) are distinct systemic anticancer treatments that are considered for different but also overlapping cancer types and stages. Although chemotherapy is an established risk factor for cancer-associated thrombosis (CAT) [1,2], emerging evidence suggests that ICIs may also increase thrombotic risk [2,3]. Independently of CAT, a prothrombotic state was associated with adverse outcomes such as treatment failure and increased mortality in cancer patients [4], highlighting the importance of understanding treatment-induced alterations in hemostasis and pathways beyond hemostasis.

Given their fundamentally different mechanism of action and the fact that these therapies are used in different patient populations, the pathophysiology underlying the development of adverse outcomes may differ between these 2 treatments. Chemotherapy has been linked to procoagulant changes driven by induction of inflammation, endothelial dysfunction, cell lysis, and alteration of hemostatic biomarkers [1,5,6]. The mechanisms by which ICIs promote hypercoagulability may involve disrupting immunoregulatory pathways, leading to excessive cytokine release, inflammation, and neutrophil extracellular trap formation [7].

Recently, we observed that longitudinal levels of a selected panel of hemostatic biomarkers (including D-dimer, soluble P-selectin, extracellular vesicle tissue factor activity, and citrullinated histone H3, a marker of neutrophilic extracellular traps) were similar between patients treated with ICIs and chemotherapy [8]. However, the contribution of other hemostatic biomarkers and of biological pathways beyond coagulation as an explanation for potentially different mechanisms of CAT or other adverse outcomes during treatment has not been evaluated to date. Until now, plasma proteomic studies in patients with cancer have primarily focused on identifying biomarkers for predicting therapy response or survival, usually within a single cancer type and treatment regimen [[9], [10], [11]]. No studies have directly compared longitudinal plasma protein profiles of patients receiving ICIs and those receiving chemotherapy, with a specific focus on potential mechanisms for CAT or other adverse outcomes during treatment. The protein profiles associated with both treatments likely differ, potentially revealing distinct biological mechanisms through which these therapies contribute to hypercoagulability and thus thrombosis. Therefore, this pilot study aimed to characterize the plasma protein profiles in patients with active non–small cell lung cancer (NSCLC) and head and neck cancer during treatment with ICI and chemotherapy, with the goal of generating hypotheses regarding potential distinct mechanisms between these treatments and regarding biomarkers associated with clinical outcomes. Specifically, we aimed to describe and compare the longitudinal changes in plasma protein profiles in patients receiving ICI or chemotherapy. Additionally, as an exploratory analysis limited by the small sample size and number of events, we assessed possible associations of protein profiles with clinical outcomes, including venous and arterial thromboembolic events (VTE and ATE), clinically relevant bleeding (CRB), all-cause mortality, and treatment failure.

## 2 Methods

### 2.1 Study population and design

A matched cohort study was conducted within the framework of the Vienna Cancer and Thrombosis and Bleeding Study (CAT-BLED), an ongoing, observational, single-center cohort study. Full details of the Vienna CAT-BLED study were previously described [12,13]. In brief, the CAT-BLED study has been enrolling patients with histologically confirmed active cancer (either newly diagnosed or recurrent/progressive disease) who are initiating systemic anticancer therapy at the Medical University of Vienna since June 2019. The study was approved by the local ethics committee (EC number: 1533/2019; ethik-kom@meduniwien.ac.at) and conducted in accordance with the Declaration of Helsinki. All participants gave written informed consent at study inclusion.

In the current analysis, we excluded patients who were receiving anticoagulation at inclusion or were exposed to prior systemic anticancer treatments (excluding prior surgery and/or radiation therapy), to avoid the influence of previous systemic anticancer treatment on the plasma protein profile. The analysis was restricted to patients with NSCLC and head and neck cancer, as these were 2 of the most common indications for ICI treatment in our population, as well as to reduce clinical and biological heterogeneity and limit confounding. Eligible patients treated with chemotherapy or ICI were matched (1:1) using exact matching for sex, cancer type, and stage (localized, lymph node involvement, or metastatic) and nearest neighbor matching for age using the R package MachIt [14]. Among 284 NSCLC patients included in the CAT-BLED study, 44 were initiating monotherapy with ICIs and 27 with chemotherapy, resulting in 10 matched pairs. Of 143 head and neck cancer patients, 22 were initiating ICIs and 11 were initiating chemotherapy, allowing for 5 matched pairs. Patients were assigned to their treatment modalities based on the prevailing treatment guidelines at the time of initiation, clinical presentation, and oncologists’ discretion. The treatment decision was made in multidisciplinary tumor boards based on tumor factors (histology, stage, expression of programmed death-ligand 1 [PD-L1], and other biomarkers), patient-related factors (comorbidities, tumor burden, performance status, and organ function), as well as patient preference.

### 2.2 Blood collection

Citrated blood samples were collected at study inclusion (ie, before initiation of systemic anticancer therapy), and subsequently 3 weeks and at 3 months after treatment initiation. Blood samples were drawn into Vacutainer citrate tubes (Vacuette; Greiner-Bio One) and immediately centrifuged for 10 minutes at 3000 × *g* at 18°C, aliquoted, and then stored at −80°C. Of the 30 patients included, all had baseline samples drawn, while 25 had samples at both the 3-week and 3-month follow-ups, resulting in a total of 80 samples. Two patients died within the first 3 months, while the other 6 samples were missing due to unsuccessful or refused blood draws.

### 2.3 Quantitative protein mass spectrometry measurements

We quantified the plasma levels of 159 proteins represented by 162 peptides using a quantitative protein mass spectrometry assay. The assay includes proteins from several functional domains, including, among others, 18 complement proteins, 14 apolipoproteins, and 21 coagulation and fibrinolytic proteins as annotated in the Human Protein Atlas (Supplementary Table 1) [15,16]. These proteins were selected based on detectability in previous studies [[17], [18], [19], [20]]. Development and validation of this quantitative protein mass spectrometry assay were previously described. In brief, in this method, plasma proteins were digested with trypsin into peptides uniquely representing the protein of interest. Sample preparation, including digestion, was automated using the BRAVO Liquid Handling System (Agilent Technologies). Peptides were purified through solid-phase extraction, separated using a reversed-phase ultra-high-performance liquid chromatography column (EclipsePlusC18 RRHD 150 × 2.1 mm, 1.8 μm particles; Agilent Technologies) and analyzed on a triple-quadrupole mass spectrometer (Agilent 6495C; Agilent Technologies) operating in multiple reaction monitoring mode. In the mass spectrometer, peptides were selected based on their mass-to-charge ratio and detected. For accurate relative quantification, synthetic stable isotope-labeled peptides, identical in sequence to the target peptides but heavier due to inclusion of ^13^C or ^15^N, were added in equal amounts to each sample as internal standards to correct for variability in sample preparation and instrument response. The ratio of endogenous to stable isotope-labeled peptide was then used to quantify the target protein. For external calibration, values were standardized to a pooled citrate plasma calibrator included in the measurement. Quality control samples measured together with study samples confirmed the assay performance, as the coefficient of variation of all proteins was comparable to previous results [21].

### 2.4 Clinical outcomes

Patients were followed for up to 2 years via regular personal follow-up visits and electronic medical record screening. All clinical outcomes were validated by an independent adjudication committee comprising experts in radiology, angiology, hematology, cardiology, and neurology. Clinical outcomes of interest included CRB, defined as major bleeding and clinically relevant nonmajor bleeding according to International Society on Thrombosis and Haemostasis criteria [22,23]; VTE, including deep vein thrombosis, pulmonary embolism, visceral vein thrombosis, catheter-related thrombosis, and symptomatic superficial vein thrombosis of the lower limb with at least 5 cm extension; ATE including ischemic stroke, myocardial infarction, and peripheral arterial occlusion; and all-cause mortality. Treatment failure was assessed by an experienced radiologist according to the immune Response Evaluation Criteria in Solid Tumors guidelines [24].

### 2.5 Statistical analysis

We performed unsupervised hierarchical clustering using the Ward minimum variance method with Euclidean distance on the scaled and centered protein concentrations at baseline to describe their association with the characteristics of patients receiving ICIs and of patients receiving chemotherapy (visualized using a heatmap). The dendrogram was cut to obtain clusters based on visual inspection of the dendrogram structure. Longitudinal changes in the protein levels over time were assessed separately for each protein through linear mixed-effects models, with an unstructured covariance matrix. Protein abundance was included as the outcome, with time, treatment group, and their interaction as fixed effects, and a random intercept for each participant to account for within-subject correlations across repeated measurements, using the baseline sample as reference. To adjust for multiple testing, the models were corrected for false discovery rate (FDR) using the Benjamini and Hochberg procedure [25]. All analyses were performed in the whole cohort and stratified by cancer type (NSCLC and head and neck cancer). Association of the baseline protein profile and the occurrence of clinical outcomes (CRB, combined end-point of VTE and ATE, all-cause mortality) and treatment failure during follow-up was assessed through the Wilcoxon rank test (with and without FDR correction) and estimation of fold-changes (FC; ie, the ratio of the mean protein levels in patients developing the adverse event to the mean levels in the patients who did not). Unsupervised clustering using the Ward minimum variance method with Euclidean distance was used to assess the association of the mean difference of the plasma protein profiles (ie, subtraction of the absolute concentrations at 3 weeks or 3 months with baseline concentration) with the occurrence of adverse events and treatment failure during follow-up. All analyses were performed with a complete-case approach using RStudio (R version 4.4.0. R Foundation for Statistical Computing, 2023: http://www.R-project.org/).

## 3 Results

### 3.1 Study population

In total, 30 matched patients were included in the present pilot study, of whom 15 received ICI and 15 received chemotherapy (10 NSCLC pairs and 5 head and neck cancer pairs). Patients treated with ICI had a median age of 75 years (interquartile range [IQR], 56-78), while patients treated with chemotherapy had a median age of 65 years (IQR, 62-78; Table). In both cohorts, 40% were male. Among the matched patients, 8 had cancer with lymph node involvement, whereas 22 (73%) presented with metastatic disease. All patients were systemically treatment-naïve at inclusion. Nearest neighbor age matching resulted in a mean intrapair age difference of 9 years (standard deviation, 5.5; range, 0-16, Supplementary Table 2). Of the 159 proteins included in the assay, 2 (calponin-1 and mucin-16) were not measurable in any of the samples and were therefore excluded from further analysis.

**Table:** Baseline characteristics of the whole matched cohort, stratified by anticancer treatment.

Patient characteristics	Overall (30)	Chemotherapy (15)	ICI (15)
Age, y, median (IQR)	66 (57-78)	65 (62-78)	75 (56-78)
BMI, kg/m^2^, median (IQR)	23.3 (20.4-26.6)	23.8 (20.4-26.9)	23.1 (20.4-26.0)
Sex, male, n (%)	12 (40)	6 (40)	6 (40)
ECOG			
0, n (%)	11 (37)	6 (40)	5 (33)
1, n (%)	14 (47)	7 (47)	7 (47)
2, n (%)	5 (16)	2 (13)	3 (20)
Cancer type			
NSCLC	20 (67)	10 (67)	10 (67)
Head and neck	10 (33)	5 (33)	5 (33)
Cancer stage			
LN involvement, n (%)	8 (27)	4 (27)	4 (27)
Metastatic disease, n (%)	22 (73)	11 (73)	11 (73)
Cancer diagnosis			
Newly diagnosed, n (%)	22 (73)	12 (80)	10 (67)
Recurrent, n (%)	8 (27)	3 (20)	5 (33)
Treatment characteristics			
Treatment-naïve, n (%)	30 (100)	15 (100)	15 (100)
Previous surgery, n (%)	10 (33)	5 (33)	5 (33)
Previous radiotherapy, n (%)	16 (53)	9 (60)	7 (47)
Antiplatelet therapy, n (%)	8 (27)	4 (27)	4 (27)
Treatment approach			
Palliative, n (%)	27 (90)	12 (80)	15 (100)
Curative, n (%)	3 (10)	3 (20)	0 (0)
ICI therapy type			
Pembrolizumab	13 (43)	-	13 (86)
Atezolizumab	1 (3)	-	1 (7)
Cemiplimab	1 (3)	-	1 (7)
Chemotherapy type			
Carboplatin/Vinorelbine	1 (3)	1 (7)	-
Carboplatin/Etoposide	1 (3)	1 (7)	-
Carboplatin/Gemcitabine	1 (3)	1 (7)	-
Carboplatin/Pemetrexed	1 (3)	1 (7)	-
Carboplatin/Paclitaxel	3 (10)	3 (20)	-
Cisplatin/Vinorelbine	4 (13)	4 (27)	-
Cisplatin/Gemcitabine	1 (3)	1 (7)	-
Cisplatin/Pemetrexed	1 (3)	1 (7)	-
Pemetrexed	2 (7)	2 (13)	-
Clinical history			
History of VTE, n (%)	0 (0)	0 (0)	0 (0)
History of ATE, n (%)	5 (17)	3 (20)	2 (13)
Laboratory parameters			
Hemoglobin, median (IQR)	13.1 (11.4-14.2)	13.1 (10.9-14.0)	12.7 (11.5-14.5)
Thrombocytes, median (IQR)	279 (252-354)	267 (252-351)	289 (247-390)
Leukocytes, median (IQR)	8.2 (7.4-11.2)	8.0 (6.5-12.2)	8.3 (7.5-10.2)

Regarding the ethnicity, all patients were of European descendance. ATE, arterial thromboembolism; BMI, body mass index; ECOG, Eastern Cooperative Oncology Group; ICI, immune checkpoint inhibitor; IQR, interquartile range; LN, lymph node; NSCLC, non–small cell lung cancer; VTE, venous thromboembolism.

### 3.2 Differences in protein profiles before anticancer treatment

**Figure 1:**
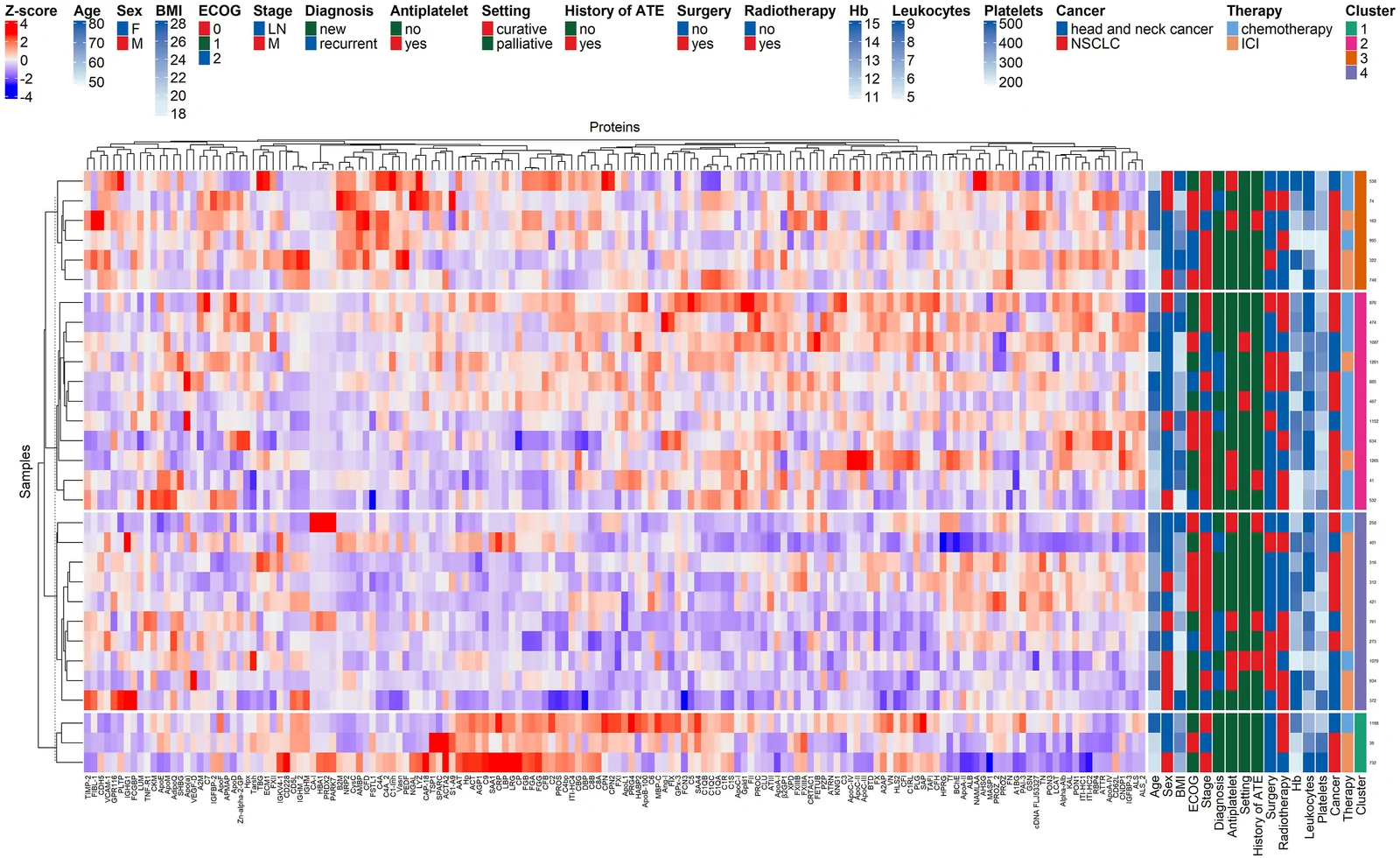
Heatmap of protein profiles at baseline, before treatment. The patients are on the rows, proteins in columns; unsupervised hierarchical clustering is shown on the top and left sides of the heatmap. Annotations on the right side show Age: on a color scale from 50 to 80 (light blue to dark blue); Sex: blue for female patients and red for male patients; Body mass index: on a color scale from 18 to 28 (light blue to dark blue); Eastern Cooperative Oncology Group performance status: red for 0, green for 1, blue for 2; Stage: blue for lymph node involvement, red for metastatic; Diagnosis: green for new diagnosis, blue for recurrent; Antiplatelet: green for no and red for yes; Setting: red for curative and green for palliative; History of arterial thromboembolism: green for no and red for yes; Previous surgery: blue for no and red for yes; Previous radiotherapy: blue for no and red for yes; hemoglobin: on a color scale from 11 to 15 (light blue to dark blue); leukocytes: on a color scale from 5 to 9 (light blue to dark blue); platelets: on a color scale from 200 to 500 (light blue to dark blue); Cancer: cancer type, blue for head and neck cancer, red for non–small cell lung cancer; Therapy: after baseline, light blue for chemotherapy and orange for ICI (immune checkpoint inhibitors); Cluster: hierarchical clustering using Ward’s minimum variance method with Euclidean distance. ATE, arterial thromboembolism; BMI, body mass index; ECOG, Eastern Cooperative Oncology Group; Hb, hemoglobin; ICI, immune checkpoint inhibitor; LN, lymph node; M, metastatic; NSCLC, non–small cell lung cancer.

At baseline (before treatment), patients’ protein profiles clustered according to treatment assignment (Figure 1), as 12 out of 15 patients receiving chemotherapy and 10 out of 15 patients receiving ICI clustered together in clusters 2 and 3 and in clusters 1 and 4, respectively. The list of the 14 most significantly different proteins between the 2 treatment groups at baseline is provided in Supplementary Table 3. Among those, proteins related to immunity (CD5 antigen-like and immunoglobulin mu chain C protein) were increased in patients with ICI treatment assignment compared with patients with chemotherapy treatment assignment (FC 1.57 for both proteins), while proteins related to coagulation were decreased (coagulation factor [F] X: FC 0.89, prothrombin: FC 0.86, kininogen-1: FC = 0.87, vitamin K-dependent protein C: FC 0.79). Within each treatment assignment group, baseline protein profile clustered by cancer type (NSCLC or head and neck cancer; Supplementary Figures 1 and 2).

### 3.3 Changes in plasma protein profiles during treatment with ICI and chemotherapy

The levels of 2 proteins changed during ICI treatment, when compared with baseline. In particular, β-2-microglobulin levels were increased at 3 weeks of treatment (mean difference of −0.13; 95% confidence interval [CI], −0.25 to −0.01), while apolipoprotein D levels were decreased at 3 months (mean difference of 0.37; 95% CI, 0.10 to 0.7; Supplementary Figures 3 and 4). These differences were not consistent across time points and did not remain after correction for FDR.

**Figure 2:**
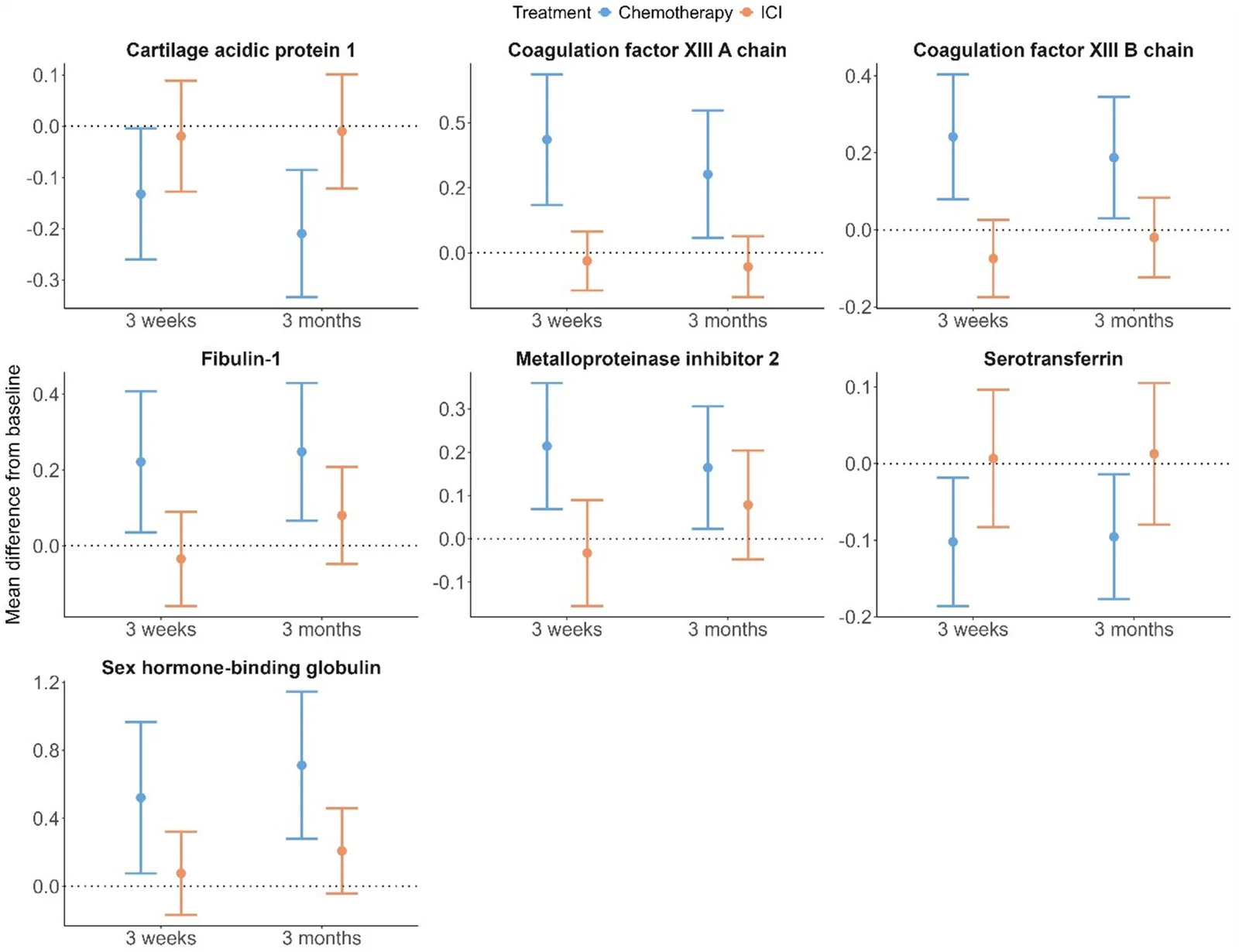
Most increased and decreased plasma proteins during chemotherapy, compared with ICI. The horizontal dotted line indicates no difference between the 2 treatments. Dots and error bars represent mean differences with 95% confidence intervals. Results for chemotherapy are shown in light blue and for ICI in orange. ICI, immune checkpoint inhibitors.

The levels of 34 (21%) proteins changed when compared with baseline in patients treated with chemotherapy, of which 12 (7%) remained after FDR correction (Supplementary Figures 3 and 4). Of these 34 proteins, 8 were associated with complement or immunity, 6 were involved in blood coagulation, and 4 were transport proteins. In particular, the levels of 7 proteins were consistently different both at 3 weeks and at 3 months (Figure 2). Of these, coagulation FXIII A and B chain (mean difference of 0.30 [95% CI, 0.06-0.54] and of 0.18 [95% CI, 0.03-0.34] at 3 months, respectively), fibulin-1 (mean difference of 0.25 [95% CI, 0.10-0.43], at 3 months), metalloproteinase inhibitor 2 (mean difference of 0.16 [95% CI, 0.02-0.31, at 3 months) and sex hormone-binding globulin (mean difference of 0.71 [95% CI, 0.23-1.14, at 3 months) were increased at both times. The levels of serotransferrin and cartilage acid protein 1 were decreased at 3 weeks and 3 months (mean difference of −0.10 [95% CI, −0.18 to −0.01] and of −0.21 [95% CI, −0.33 to −0.09] at 3 months, respectively). Results were overall similar when stratified by cancer type (Supplementary Figures 5–8).

### 3.4 Association of protein profiles with the occurrence of adverse events

**Figure 3:**
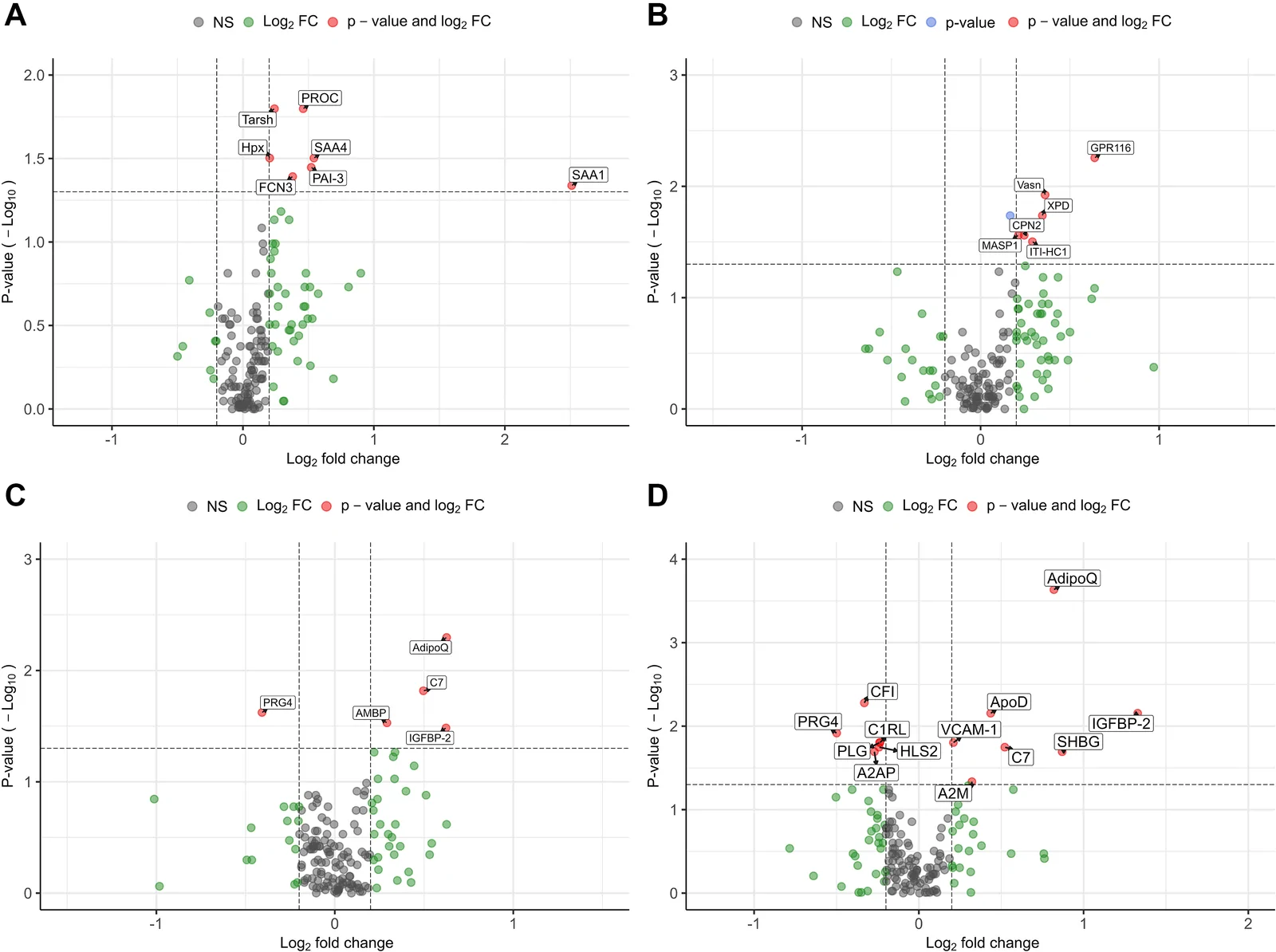
Association of baseline protein levels with clinical outcomes during follow-up: (A) venous or arterial events, (B) major or clinically relevant non-major bleeding, (C) mortality, and (D) treatment failure. In this plot, each dot represents a protein. The fold-change on the x-axis (ratio of the mean baseline protein level in patients experiencing a clinical outcome to that in the patients who did not) is plotted against the significance of this difference on the y-axis (expressed as -log10 of the *P* value from the Wilcoxon rank-sum test). The most significant proteins appear at the top of the plots. Protein levels are increased (left side) or decreased (right side) at baseline in patients who later experienced a clinical outcome during follow-up compared with patients who did not. FC, fold change; NS, not significant.

During a median follow-up of 400 days (IQR, 283-1036), 5 patients developed a VTE and one developed an ATE (after a median of 175 days; IQR, 55-215). Moreover, 6 patients experienced CRB (after a median of 38 days; IQR, 20-170). Treatment failure occurred in 22 patients and, overall, 17 patients died during follow-up. In our exploratory analysis, we observed that, at baseline, levels of 7 proteins were elevated in patients later developing a venous or an arterial event (Figure 3). In particular, levels of serum amyloid A-1, A-2 and A-4, vitamin K-dependent protein C and plasma serine protease inhibitor were > 30% increased at baseline in the patients developing a VTE or ATE during follow-up compared to those who did not. Baseline levels of 6 proteins (serum paraoxonase/arylesterase 1, mannan-binding lectin serine protease 1, carboxypeptidase N subunit 2, inter-α-trypsin inhibitor heavy chain H1, Xaa-Pro dipeptidase, vasorin, adhesion G protein-coupled receptor F5) were elevated in patients who subsequently experienced bleeding (Figure 3). Moreover, baseline levels of 5 and 14 proteins were associated with mortality and treatment failure, respectively (Figure 3). After correction for multiple testing, only elevated levels of adiponectin at baseline remained associated with treatment failure. At 3 months, patients who developed bleeding showed a decrease in protein levels compared with their baseline measurements, whereas patients who did not develop bleeding events showed relatively stable levels (Supplementary Figure 9). In particular, levels of complement component C7, adhesion G protein-coupled receptor F5, clusterin, and vitamin K-dependent protein C were more markedly decreased at 3 months in patients who experienced a bleeding event compared with those who did not. No clustering was observed based on the occurrence of other adverse events (VTE or ATE, treatment failure or mortality).

## 4 Discussion

In this pilot study, we investigated baseline and on-treatment plasma protein profiles of patients with NSCLC and head and neck cancer receiving either monotherapy with ICIs or chemotherapy. Our findings suggest distinct protein profiles, both across patients before treatment initiation and within patients during therapy, highlighting the value of assessing plasma protein levels longitudinally to elucidate treatment-specific effects and identify potential biomarkers associated with clinical outcomes. However, given the exploratory nature of the study and the small sample size, our findings should be interpreted as hypothesis-generating and are intended to inform future validation in larger cohorts rather than establish treatment-specific biological effects or biomarkers.

Already at baseline, before systemic anticancer therapy was initiated, plasma proteins clustered according to treatment assignment. Notably, proteins associated with immune responses were elevated in the patients who would later receive ICIs, whereas coagulation-related proteins were decreased compared with patients later receiving chemotherapy. These differences are not unexpected as the anticancer treatment was not randomized. For instance, patients deemed unfit for chemotherapy were more likely to receive ICIs only while a low PD-L1 tumor expression does not favor ICI monotherapy [26,27]. Patients well suited for ICI therapy typically harbor T-cell-inflamed, PD-L1-positive cancers [28]. In this setting, PD-L1 expression is widely considered to be induced by interferon-γ as well as other inflammatory mediators released by tumor-infiltrating T lymphocytes [[29], [30], [31]]. By contrast, patients with a higher symptomatic burden, more aggressive or bulky disease, and a clinical need for rapid tumor debulking, and who are fit enough for chemotherapy, are more likely to receive chemotherapy [32]. Such biologically highly active tumors are strongly associated with a pronounced activation of hemostasis [33,34]. These mechanisms provide a plausible explanation why, already at baseline, patients allocated to ICI therapy show higher levels of immune-response proteins, whereas those selected for chemotherapy have higher circulating concentrations of coagulation-related proteins.

During treatment, we observed minimal changes in plasma protein levels in patients receiving ICIs. Only 2 proteins (β-2-microglobulin and apolipoprotein D) showed alterations, and these dissolved after correction for multiple testing. By contrast, plasma concentrations of 34 proteins changed during chemotherapy. Most of these proteins were involved in complement pathways, regulation of immunity, blood coagulation or had transport functions. As chemotherapy interferes with fundamental cellular processes [1], the resulting systemic inflammatory and repair responses are likely reflected in the pronounced shifts in circulating protein levels. In contrast, ICIs primarily modulate immune-checkpoint signaling and reinvigorate T-cell responses [7], which may lead to more subtle changes in the circulating proteome that are largely restricted to immune-related pathways, consistent with the comparatively stable protein profiles observed in the ICI-treated group. Notably, our protein panel does not include known ICI responsive chemokines (eg, CXCL9, CXCL10, CXCL 11) and cytokines (eg, interleukins and tumor necrosis factors), which have been shown to increase early after treatment [35,36]. The lack of changes in the remaining inflammatory and immune-related markers present in our panel may reflect limited statistical power, inter-individual variability, or the timing of sampling, which may miss transient early immune activation in the first days of treatment.

Notably, levels of coagulation FXIII A and B chain showed the largest increases during active chemotherapy treatment in our cohort. Factor XIII is a transglutaminase that is activated by thrombin, a central enzyme in the coagulation cascade [37]. Once activated, it cross-links fibrin strands, thereby stabilizing the clot [37]. A previous study similarly reported elevated FXIII A chain levels in patients with NSCLC during chemotherapy, and FXIII A chain levels >150% were independently associated with treatment failure, although most patients in that cohort received combination regimens including ICIs [38]. Additionally, higher FXIII activity has been observed in patients with advanced-stage NSCLC compared with healthy controls and those with early-stage disease [39]. By contrast, reduced FXIII levels have been reported during consolidation chemotherapy in patients with acute leukemia, and these decreases were directly related to bleeding events, suggesting that chemotherapy-induced changes in FXIII are both cancer- and context-dependent [40]. Further studies in larger cancer cohorts are needed to define the determinants and prognostic significance of FXIII changes during anticancer therapy and to clarify its potential role as a hemostatic and prognostic biomarker for thrombosis, bleeding, and treatment failure.

We observed that elevated levels of specific proteins were associated with the occurrence of clinical outcomes during the follow-up. In particular, patients who subsequently developed VTE or ATE, experienced bleeding, died, or had treatment failure showed higher baseline levels of several inflammatory and hemostatic proteins compared with those without these events. Among these, adiponectin and complement component C7 were associated with death and treatment failure; heparin cofactor II and sex hormone-binding globulin were associated with treatment failure; and ficolin-3 was associated with VTE or ATE. Notably, all of these proteins were among those that changed during chemotherapy. Interestingly, despite the observed changes in other coagulation-related proteins during chemotherapy, including FXIII A and B, only baseline levels heparin cofactor II was associated with clinical outcomes in our cohort. In addition, changes in complement (eg, complement component C7 and clusterin) or hemostatic (eg, vitamin K-dependent protein C) proteins were more pronounced in patients experiencing bleeding. Additionally, patients who suffered from a bleeding event showed a decrease in protein levels when compared to the baseline, which is consistent with a previous observation in the same cohort in which low albumin levels were associated with an increased risk of bleeding [13]. However, due to the small number of patients included in the present study, these analysis were only exploratory, and these results could not be stratified by treatment type. Therefore, as expected, none of these differences remained after correction for multiple testing, except for the association of elevated adiponectin levels with treatment failures.

Adiponectin is an adipokine involved in the regulation of glucose metabolism and fatty acid oxidation [41]. Altered adiponectin levels have been reported in cancer-associated cachexia, a pathological state characterized by profound weight loss due to skeletal muscle wasting and depletion of adipose tissue stores [42]. The relation between adiponectin and cachectic state might thus explain its association with mortality and treatment failure observed in our study. Previous studies have described increased adiponectin concentrations in patients with cachexia and coexisting malignancies, although other studies have found no significant differences between cachectic and non-cachectic patients [43,44]. Further studies will be needed to explore adiponectin as a potential marker of cachexia, treatment failure, and mortality in patients with cancer.

In this pilot study, we were able to quantify more than 100 proteins in a small volume of plasma (<10 μL). To isolate the effect of each treatment, we addressed the issue of confounding by indication through a careful matching procedure. However, our results should be interpreted considering several limitations. First, the exploratory nature of the analyses and the relatively small sample size (a consequence of the strict matched study design) limited the ability to detect modest differences between the 2 treatment groups and potentially resulted in instability of the hierarchical clustering analyses. The sample size was determined with the aim of isolating the treatment effect while minimizing confounding as much as possible. However, although we matched patients on age, sex, cancer type, and stage to isolate treatment-related differences, residual confounding by indication cannot be excluded, as we were unable to fully account for factors such as prior surgery or radiation treatment, Eastern Cooperative Oncology Group performance status, comorbidities, or frailty. Additionally, the head and neck cancer group in our study is histologically heterogeneous, and we were unable to account for these biological differences in our analysis due to the limited sample size. Second, proteomic data were unavailable for 10 missing plasma samples; however, no patients were lost to follow-up, only 2 deaths occurred within the first 3 months after inclusion, and missing samples were caused by unsuccessful blood draws or patient refusal, which is unlikely to have introduced systematic bias. Third, selection bias may have arisen from including only patients for whom a suitable matched counterpart receiving the alternative treatment was available. Fourth, given that some events occurred after the last protein measurement at 3 months, associations with the protein profiles may have been influenced by time-varying confounding during treatment or attenuated by regression dilution bias. Finally, all patients were treated at a single tertiary university hospital and were of European descent, and indications for ICIs and chemotherapy, patterns of health care access, and institutional prescribing practices are evolving rapidly, limiting the generalizability of our findings.

In conclusion, our pilot study suggests treatment-specific changes in plasma protein profiles between patients with head and neck cancer and NSCLC receiving ICI and chemotherapy. These findings highlight the potential of longitudinal protein profiling to generate hypotheses regarding treatment-specific effects and candidate biomarkers associated with clinical outcomes. Validation of these exploratory associations in larger, independent cohorts is warranted to further assess the clinical utility of the identified protein profiles for understanding the risk of thrombosis, bleeding, treatment failure, and mortality in patients with cancer.
