# Supplementary material for: Distinct changes in plasma protein profiles of cancer patients receiving immune checkpoint inhibitors versus chemotherapy: implications for thrombosis risk and adverse outcomes
Source: Res Pract Thromb Haemost. 2026 Aug 27;10(6):106929. doi: 10.1016/j.rpth.2026.106929 (PMC13602718; doi:10.1016/j.rpth.2026.106929)
Supplement: Supplementary file 1 [file mmc1.docx]

**Supplementary Material for: Distinct changes in plasma protein profiles of cancer patients receiving immune checkpoint inhibitors versus chemotherapy: implications for thrombosis risk and adverse outcomes**

**Table of contents**

[Supplementary Table 1. Overview of the 159 measured proteins, their representative peptide and secretome function as annotated by the Human Protein atlas.	2](#_Toc231985533)

[Supplementary Table 2. Matched patients and covariates included in the matching	7](#_Toc231985534)

[Supplementary Table 3. Protein levels different at baseline (before treatment) comparing patients treated with ICI and chemotherapy	8](#_Toc231985535)

[Supplementary Figure 1. Heatmap of protein profiles at baseline in patients treated with ICI.	9](#_Toc231985536)

[Supplementary Figure 2. Heatmap of protein profiles at baseline in patients treated with chemotherapy.	10](#_Toc231985537)

[Supplementary Figure 3. Mean differences in protein levels that decreased during chemotherapy or ICI compared with baseline	11](#_Toc231985538)

[Supplementary Figure 4. Mean differences in protein levels that increased during chemotherapy or ICI compared with baseline.	12](#_Toc231985539)

[Supplementary Figure 5. Mean differences in protein levels that decreased during chemotherapy in patients with NSCLC compared with baseline.	13](#_Toc231985540)

[Supplementary Figure 6. Mean differences in protein levels that increased during chemotherapy in patients with NSCLC compared with baseline.	14](#_Toc231985541)

[Supplementary Figure 7. Mean differences in protein levels that decreased during chemotherapy in patients with head and neck cancer compared with baseline.	15](#_Toc231985542)

[Supplementary Figure 8. Mean differences in protein levels that increased during chemotherapy in patients with head and neck cancer compared with baseline.	16](#_Toc231985543)

[Supplementary Figure 9. Heatmap depicting mean changes in protein levels between baseline and three months, and their associations with clinical outcomes during follow-up.	17](#_Toc231985544)

Supplementary Table 1. Overview of the 159 measured proteins, their representative peptide and secretome function as annotated by the Human Protein atlas. Proteins with * are incorporated in the system suitability mix measured before and after each run.

| **Protein** | **Peptide** | **UniProtKB Accession Number** | **Protein abbreviation** | **Secretome function** |
| --- | --- | --- | --- | --- |
| Alpha-1-acid glycoprotein 1 | NWGLSVYADKPETTK | P02763 | AGP1 | Acute phase |
| Alpha-1-antichymotrypsin | EIGELYLPK | P01011 | ACT | Acute phase |
| Alpha-2-antiplasmin | LGNQEPGGQTALK | P08697-1 | A2AP | Acute phase |
| C-reactive protein | AFVFPK | P02741 | CRP | Acute phase |
| Fibronectin | HTSVQTTSSGSGPFTDVR | P02751 | FN | Acute phase |
| Haptoglobin | DIAPTLTLYVGK | P00738 | Hp | Acute phase |
| Inter-alpha-trypsin inhibitor heavy chain H4 | SPEQQETVLDGNLIIR | Q14624 | ITI-HC4 | Acute phase |
| Serum amyloid A-1 and A-2 proteins | EANYIGSDK | P0DJI8 | SAA1 | Acute phase |
| Serum amyloid A-4 protein | GNYDAAQR | P35542 | SAA4 | Acute phase |
| Apolipoprotein A-I | ATEHLSTLSEK | P02647 | ApoA-I | Apolipoprotein |
| Apolipoprotein A-II | SPELQAEAK | P02652 | ApoA-II | Apolipoprotein |
| Apolipoprotein A-IV | LGEVNTYAGDLQK | P06727 | ApoA-IV | Apolipoprotein |
| Apolipoprotein B-100 | FPEVDVLTK | P04114 | ApoB-100 | Apolipoprotein |
| Apolipoprotein C-I | EWFSETFQK | P02654 | ApoC-I | Apolipoprotein |
| Apolipoprotein C-II | TYLPAVDEK | P02655 | ApoC-II | Apolipoprotein |
| Apolipoprotein C-III | GWVTDGFSSLK | P02656 | ApoC-III | Apolipoprotein |
| Apolipoprotein C-IV | ELLETVVNR | P55056 | ApoC-IV | Apolipoprotein |
| Apolipoprotein D | NILTSNNIDVK | P05090 | ApoD | Apolipoprotein |
| Apolipoprotein E | LGPLVEQGR | P02649 | ApoE | Apolipoprotein |
| Apolipoprotein F | SGVQQLIQYYQDQK | Q13790 | ApoF | Apolipoprotein |
| Apolipoprotein L1 | VAQELEEK | O14791 | ApoL1 | Apolipoprotein |
| Apolipoprotein M | AFLLTPR | O95445 | ApoM | Apolipoprotein |
| Beta-2-glycoprotein 1 | ATVVYQGER | P02749 | β2GP1 | Apolipoprotein |
| Alpha-1-antitrypsin | SVLGQLGITK | P01009 | AAT | Blood coagulation |
| Antithrombin | DDLYVSDAFHK | P01008 | ATIII | Blood coagulation |
| Coagulation factor IX * | SALVLQYLR | P00740 | FIX | Blood coagulation |
| Coagulation factor X | MLEVPYVDR | P00742 | FX | Blood coagulation |
| Coagulation factor XI | TSESGLPSTR | P03951 | FXI | Blood coagulation |
| Coagulation factor XII | EQPPSLTR | P00748 | FXII | Blood coagulation |
| Coagulation factor XIII A chain | GTYIPVPIVSELQSGK | P00488 | FXIIIA | Blood coagulation |
| Coagulation factor XIII B chain | IQTHSTTYR | P05160 | FXIIIB | Blood coagulation |
| Fibrinogen alpha chain | VQHIQLLQK | P02671 | FGA | Blood coagulation |
| Fibrinogen beta chain | HQLYIDETVNSNIPTNLR | P02675 | FGB | Blood coagulation |
| Fibrinogen gamma chain | YEASILTHDSSIR | P02679 | FGG | Blood coagulation |
| Heparin cofactor 2 | TLEAQLTPR | P05546 | HLS2 | Blood coagulation |
| Histidine-rich glycoprotein | ADLFYDVEALDLESPK | P04196 | HPRG | Blood coagulation |
| Kininogen-1 | DIPTNSPELEETLTHTITK | P01042 | KNG1 | Blood coagulation |
| Plasma protease C1 inhibitor * | FQPTLLTLPR | P05155 | C1-Inh | Blood coagulation |
| Plasminogen | EAQLPVIENK | P00747 | PLG | Blood coagulation |
| Prothrombin | ELLESYIDGR | P00734 | FII | Blood coagulation |
| Thrombin-activatable fibrinolysis inhibitor | IAWHVIR | Q96IY4 | TAFI | Blood coagulation |
| Vitamin K-dependent protein C | LGEYDLR | P04070 | PROC | Blood coagulation |
| Vitamin K-dependent protein S | VYFAGFPR | P07225 | PROS | Blood coagulation |
| Vitamin K-dependent protein Z | DFAEHLLIPR | P22891 | PROZ | Blood coagulation |
| Vitamin K-dependent protein Z | GLLSGWAR | P22891 | PROZ | Blood coagulation |
| Insulin-like growth factor-binding protein complex acid labile subunit | LAELPADALGPLQR | P35858 | ALS | Cell adhesion |
| Insulin-like growth factor-binding protein complex acid labile subunit * | NLIAAVAPGAFLGLK | P35858 | ALS | Cell adhesion |
| Thrombospondin-1 | GTLLALER | P07996 | TSP1 | Cell adhesion |
| Vascular cell adhesion protein 1 | NTVISVNPSTK | P19320 | VCAM-1 | Cell adhesion |
| Vitronectin | FEDGVLDPDYPR | P04004 | VN | Cell adhesion |
| C4b-binding protein alpha chain | EDVYVVGTVLR | P04003 | C4bp | Complement pathway |
| Clusterin | ELDESLQVAER | P10909 | CLU | Complement pathway |
| Complement C1q subcomponent subunit A | PAFSAIR | P02745 | C1QA | Complement pathway |
| Complement C1q subcomponent subunit B * | IAFSATR | P02746 | C1QB | Complement pathway |
| Complement C1q subcomponent subunit C | FQSVFTVTR | P02747 | C1QC | Complement pathway |
| Complement C1r subcomponent | GLTLHLK | P00736 | C1R | Complement pathway |
| Complement C1r subcomponent-like protein | VVVHPDYR | Q9NZP8 | C1RL | Complement pathway |
| Complement C1s subcomponent | TNFDNDIALVR | P09871 | C1S | Complement pathway |
| Complement C2 | HAFILQDTK | P06681 | C2 | Complement pathway |
| Complement C3 | TGLQEVEVK | P01024 | C3 | Complement pathway |
| Complement C4 | VGDTLNLNLR | P0C0L4 | C4A | Complement pathway |
| Complement C4 | VLSLAQEQVGGSPEK | P0C0L4 | C4A | Complement pathway |
| Complement C5 | VFQFLEK | P01031 | C5 | Complement pathway |
| Complement component C6 | DLHLSDVFLK | P13671 | C6 | Complement pathway |
| Complement component C7 | AASGTQNNVLR | P10643 | C7 | Complement pathway |
| Complement component C8 alpha chain | MESLGITSR | P07357 | C8A | Complement pathway |
| Complement component C8 beta chain | SDLEVAHYK | P07358 | C8B | Complement pathway |
| Complement component C9 | LSPIYNLVPVK | P02748 | C9 | Complement pathway |
| Complement factor I | VFSLQWGEVK | P05156 | CFI | Complement pathway |
| Extracellular matrix protein 1 | NVALVSGDTENAK | Q16610 | ECM1 | Developmental protein |
| Beta-Ala-His dipeptidase | ALEQDLPVNIK | Q96KN2 | CNDP1 | Enzyme |
| Biotinidase | SHLIIAQVAK | P43251 | BTD | Enzyme |
| Carboxypeptidase N catalytic chain | SIPQVSPVR | P15169 | CPN | Enzyme |
| Ceruloplasmin | IYHSHIDAPK | P00450 | CP | Enzyme |
| Cholinesterase * | YLTLNTESTR | P06276 | BChE | Enzyme |
| Glutathione peroxidase 3 | QEPGENSEILPTLK | P22352 | GPx-3 | Enzyme |
| Hyaluronan-binding protein 2 | VVLGDQDLK | Q14520 | HABP2 | Enzyme |
| Lysozyme C | AWVAWR | P61626 | LYZ | Enzyme |
| Phosphatidylcholine-sterol acyltransferase | SSGLVSNAPGVQIR | P04180 | LCAT | Enzyme |
| Phosphatidylinositol-glycan-specific phospholipase D | FGSSLITVR | P80108-1 | Gpld1 | Enzyme |
| Protein AMBP | HHGPTITAK | P02760 | AMBP | Enzyme |
| Serum paraoxonase/arylesterase 1 | IFFYDSENPPASEVLR | P27169 | PON1 | Enzyme |
| Serum paraoxonase/lactonase 3 | ILIGTVFHK | Q15166 | PON3 | Enzyme |
| Alpha-2-macroglobulin | AIGYLNTGYQR | P01023 | A2M | Enzyme inhibitor |
| Cystatin-C | ALDFAVGEYNK | P01034 | CysC | Enzyme inhibitor |
| Fetuin-B | LVVLPFPK | Q9UGM5 | FETUB | Enzyme inhibitor |
| Inter-alpha-trypsin inhibitor heavy chain H1 | GSLVQASEANLQAAQDFVR | P19827 | ITI-HC1 | Enzyme inhibitor |
| Inter-alpha-trypsin inhibitor heavy chain H2 | SLAPTAAAK | P19823 | ITI-HC2 | Enzyme inhibitor |
| Kallistatin | VGSALFLSHNLK | P29622 | KAL | Enzyme inhibitor |
| Metalloproteinase inhibitor 2 | EYLIAGK | P16035 | TIMP-2 | Enzyme inhibitor |
| Pregnancy zone protein | ISEITNIVSK | P20742 | PZP | Enzyme inhibitor |
| Vascular endothelial growth factor D | DLIQHPK | O43915 | VEGF-D | Growth factor |
| Adiponectin | IFYNQQNHYDGSTGK | Q15848 | AdipoQ | Hormone |
| Transthyretin | GSPAINVAVHVFR | P02766 | ATTR | Hormone |
| Beta-2-microglobulin | VNHVTLSQPK | P61769 | B2M | Immunity |
| Cathelicidin antimicrobial peptide | AIDGINQR | P49913 | CAP-18 | Immunity |
| CD5 antigen-like | LVGGLHR | O43866 | CD5L | Immunity |
| Complement factor B | EELLPAQDIK | P00751-1 | CFB | Immunity |
| Complement factor D | THHDGAITER | P00746 | CFD | Immunity |
| Complement factor H * | SSQESYAHGTK | P08603 | CFH | Immunity |
| Ficolin-3 | YAVSEAAAHK | O75636 | FCN3 | Immunity |
| Ig gamma-1 chain C region | GPSVFPLAPSSK | P01857 | IGHG1 | Immunity |
| Ig mu chain C region * | GFPSVLR | P01871 | IGHM | Immunity |
| Ig mu heavy chain disease protein and Ig mu chain C * | VSVFVPPR | P04220 | IGHM | Immunity |
| Immunoglobulin kappa variable 4-1 | NYLAWYQQKPGQPPK | P06312 | IGKV4-1 | Immunity |
| Mannan-binding lectin serine protease 1 | TGVITSPDFPNPYPK | P48740 | MASP1 | Immunity |
| N-acetylmuramoyl-L-alanine amidase | AGLLRPDYALLGHR | Q96PD5 | NAMLAA | Immunity |
| Protein S100-A9 | DLQNFLK | P06702 | S1-A9 | Immunity |
| Alpha-2-HS-glycoprotein | FSVVYAK | P02765 | AHSG | Other |
| Angiotensinogen | ALQDQLVLVAAK | P01019 | Ang-I | Other |
| Fibulin-1 | TGYYFDGISR | P23142 | FIBL-1 | Other |
| Follistatin-related protein 1 | YVQELQK | Q12841 | FSTL1 | Other |
| Gelsolin | AGALNSNDAFVLK | P06396 | GSN | Other |
| Insulin-like growth factor-binding protein 2 | LIQGAPTIR | P18065 | IGFBP-2 | Other |
| Insulin-like growth factor-binding protein 3 * | FLNVLSPR | P17936 | IGFBP-3 | Other |
| Attractin | SVNNVVVR | O75882 | ATRN | Receptor |
| Neuropilin-2 | ALQVVR | O60462 | NRP2 | Receptor |
| Tumor necrosis factor receptor superfamily member 1A | EATLELLGR | P19438 | TNF-R1 | Receptor |
| Afamin | DADPDTFFAK | P43652 | Alpha-Alb | Transport |
| Apolipoprotein(a) * | GTYSTTVTGR | P08519 | Apo(a) | Transport |
| Corticosteroid-binding globulin | WSAGLTSSQVDLYIPK | P08185 | CBG | Transport |
| Hemopexin | NFPSPVDAAFR | P02790 | Hpx | Transport |
| Lipopolysaccharide-binding protein | ITLPDFTGDLR | P18428 | LBP | Transport |
| Melanotransferrin | YYDYSGAFR | P08582 | CD228 | Transport |
| Neutrophil gelatinase-associated lipocalin | ITLYGR | P80188 | NGAL | Transport |
| Phospholipid transfer protein | AVEPQLQEEER | P55058 | PLTP | Transport |
| Plasma serine protease inhibitor | GFQQLLQELNQPR | P05154 | PAI-3 | Transport |
| Retinol-binding protein 4 | YWGVASFLQK | P02753 | RBP4 | Transport |
| Serotransferrin | DGAGDVAFVK | P02787 | Tf | Transport |
| Serum albumin | LVNEVTEFAK | P02768 | ALB | Transport |
| Sex hormone-binding globulin | TSSSFEVR | P04278 | SHBG | Transport |
| Thyroxine-binding globulin | AVLHIGEK | P05543 | TBG | Transport |
| Vitamin D-binding protein | VLEPTLK | P02774 | DBP | Transport |
| Alpha-1B-glycoprotein | LETPDFQLFK | P04217-1 | A1BG | No annotated function |
| Carboxypeptidase N subunit 2 | AGGSWDLAVQER | P22792 | CPN2 | No annotated function |
| Cartilage acidic protein 1 | GVASLFAGR | Q9NQ79 | CRTAC1 | No annotated function |
| Leucine-rich alpha-2-glycoprotein | DLLLPQPDLR | P02750 | LRG | No annotated function |
| Lumican | SLEDLQLTHNK | P51884 | LUM | No annotated function |
| Mannose-binding protein C | WLTFSLGK | P11226 | MBP-C | No annotated function |
| Mucin-16 | ELGPYTLDR | Q8WXI7 | MUC-16 | No annotated function |
| Pigment epithelium-derived factor | LQSLFDSPDFSK | P36955 | PEDF | No annotated function |
| Proteoglycan 4 | DQYYNIDVPSR | Q92954 | PRG4 | No annotated function |
| Serum amyloid P-component | IVLGQEQDSYGGK | P02743 | SAP | No annotated function |
| SPARC | LEAGDHPVELLAR | P09486 | SPARC | No annotated function |
| Target of Nesh-SH3 | IYLSDSLTGK | Q7Z7G0 | Tarsh | No annotated function |
| Tetranectin | NWETEITAQPDGGK | P05452 | TN | No annotated function |
| Vasorin | YLQGSSVQLR | Q6EMK4 | Vasn | No annotated function |
| Zinc-alpha-2-glycoprotein | EIPAWVPFDPAAQITK | P25311 | Zn-alpha-2-GP | No annotated function |
| Actin, aortic smooth muscle | SYELPDGQVITIGNER | P62736 | ACTA2 | No annotated function |
| Adhesion G protein-coupled receptor F5 | DVIVHPLPLK | Q8IZF2 | GPR116 | No annotated function |
| Adipocyte plasma membrane-associated protein | LLEYDTVTR | Q9HDC9 | APMAP | No annotated function |
| Cadherin-5 | ELDSTGTPTGK | P33151 | CDH5 | No annotated function |
| Calponin-1 | VNVGVK | P51911 | CNN1 | No annotated function |
| Carbonic anhydrase 1 | VLDALQAIK | P00915 | CA-I | No annotated function |
| cDNA FLJ53327 | EGGQTAPASTR | B7Z2X4 | cDNA FLJ53327 | No annotated function |
| Creatine kinase M-type | FEEILTR | P06732 | CKM | No annotated function |
| Hemoglobin subunit alpha | VGAHAGEYGAEALER | P69905 | HBA1 | No annotated function |
| IgGFc-binding protein | GATTSPGVYELSSR | Q9Y6R7 | FCGBP | No annotated function |
| L-selectin | AEIEYLEK | P14151 | CD62L | No annotated function |
| Peroxiredoxin-2 | GLFIIDGK | P32119-1 | PRDX2 | No annotated function |
| Protein deglycase DJ-1 | ALVILAK | Q99497 | PARK7 | No annotated function |
| Xaa-Pro dipeptidase | AVYEAVLR | P12955 | XPD | No annotated function |

| Supplementary Table 2. Matched patients and covariates included in the matching | | | | | | | | |
| --- | --- | --- | --- | --- | --- | --- | --- | --- |
|  | **ICI (15)** | | | **Chemotherapy (15)** | | |  |  |
| **Pair** | **Age** | **Gender** | **Stage** | **Age** | **Gender** | **Stage** | **Age difference** | **Cancer type** |
| 1 | 54 | F | Lymph node involvement | 63 | F | Lymph node involvement | 9 | NSCLC |
| 2 | 65 | M | Distant metastatic | 78 | M | Distant metastatic | 13 | NSCLC |
| 3 | 53 | F | Distant metastatic | 56 | F | Distant metastatic | 3 | NSCLC |
| 4 | 76 | F | Distant metastatic | 71 | F | Distant metastatic | 5 | NSCLC |
| 5 | 56 | F | Distant metastatic | 57 | F | Distant metastatic | 1 | NSCLC |
| 6 | 77 | F | Distant metastatic | 82 | F | Distant metastatic | 5 | NSCLC |
| 7 | 52 | M | Distant metastatic | 67 | M | Distant metastatic | 15 | NSCLC |
| 8 | 65 | M | Distant metastatic | 79 | M | Distant metastatic | 14 | NSCLC |
| 9 | 57 | F | Distant metastatic | 57 | F | Distant metastatic | 0 | NSCLC |
| 10 | 78 | F | Lymph node involvement | 64 | F | Lymph node involvement | 14 | NSCLC |
| 11 | 75 | M | Distant metastatic | 68 | M | Distant metastatic | 7 | Head and neck |
| 12 | 78 | M | Lymph node involvement | 62 | M | Lymph node involvement | 16 | Head and neck |
| 13 | 79 | F | Lymph node involvement | 63 | F | Lymph node involvement | 16 | Head and neck |
| 14 | 84 | M | Distant metastatic | 80 | M | Distant metastatic | 4 | Head and neck |
| 15 | 76 | F | Distant metastatic | 65 | F | Distant metastatic | 11 | Head and neck |
| Matching was performed using propensity score matching, with the following parameters: stage (equal), gender (equal) age (nearest possible). | | | | | | | | |

| Supplementary Table 3. Protein levels different at baseline (before treatment) comparing patients treated with ICI and chemotherapy | | | |  |
| --- | --- | --- | --- | --- |
| **Protein** | **Fold change*** | **P-value** | **Secretome function** | |
| Apolipoprotein L1 | 0.79 | 0.01 | Apolipoprotein | |
| CD5 antigen-like | 1.57 | 0.01 | Immunity | |
| Ig mu chain C region | 1.57 | 0.01 | Immunity | |
| Beta-2-glycoprotein 1 | 0.83 | 0.02 | Receptor | |
| Ig mu heavy chain disease protein and Ig mu chain C | 1.65 | 0.02 | Immunity | |
| Angiotensinogen | 0.87 | 0.02 | Immunity | |
| Apolipoprotein C-I | 0.67 | 0.03 | Apolipoprotein | |
| Coagulation factor X | 0.89 | 0.03 | Blood coagulation | |
| Hyaluronan-binding protein 2 | 0.90 | 0.03 | Enzyme | |
| Prothrombin | 0.86 | 0.03 | Blood coagulation | |
| Retinol-binding protein 4 | 0.67 | 0.04 | Transport | |
| Kininogen-1 | 0.87 | 0.05 | Blood coagulation | |
| Complement C5 | 0.93 | 0.05 | Complement pathway | |
| Vitamin K-dependent protein C | 0.79 | 0.05 | Blood coagulation | |
| *Fold-change represents the ratio of the mean protein levels in patients treated with ICIs to the mean levels in the patients treated with chemotherapy. A fold change > 1 indicate that the protein levels were increased in patients treated with ICI compared with patients treated with chemotherapy, while a fold change < 1 indicates decreased protein levels in ICI patients compared with chemotherapy. ICI: immune checkpoint inhibitors | | | |  |

Supplementary Figure 1. Heatmap of protein profiles at baseline in patients treated with ICI. The patients are on rows, proteins in columns, unsupervised hierarchal clustering is shown on the top and left sides of the heatmap. Annotations on the right side show Age: on a color scale from 50 to 80 (light blue to dark blue); Sex: blue for female patients and red for male patients; Body mass index (BMI): on a color scale from 18 to 28 (light blue to dark blue); Eastern Cooperative Oncology Group (ECOG) performance status: red for 0, green for 1, blue for 2; Stage: blue for lymph node involvement (LN), red for metastatic (M); Diagnosis: green for new diagnosis, blue for recurrent; Antiplatelet: green for no and red for yes; Setting: red for curative and green for palliative; History of arterial thromboembolism (ATE): green for no and red for yes; Previous surgery: blue for no and red for yes; Previous radiotherapy: blue for no and red for yes; Hemoglobin (Hb): on a color scale from 11 to 15 (light blue to dark blue); Leukocytes: on a color scale from 5 to 9 (light blue to dark blue); Platelets: on a color scale from 200 to 500 (light blue to dark blue); Cancer: cancer type, blue for head and neck cancer, red for non-small cell lung cancer (NSCLC); Cluster: hierarchical clustering using Ward’s minimum variance method with Euclidean distance.

Supplementary Figure 2. Heatmap of protein profiles at baseline in patients treated with chemotherapy. The patients are on rows, proteins in columns, unsupervised hierarchal clustering is shown on the top and left sides of the heatmap. Annotations on the right side show Age: on a color scale from 50 to 80 (light blue to dark blue); Sex: blue for female patients and red for male patients; Body mass index (BMI): on a color scale from 18 to 28 (light blue to dark blue); Eastern Cooperative Oncology Group (ECOG) performance status: red for 0, green for 1, blue for 2; Stage: blue for lymph node involvement (LN), red for metastatic (M); Diagnosis: green for new diagnosis, blue for recurrent; Antiplatelet: green for no and red for yes; Setting: red for curative and green for palliative; History of arterial thromboembolism (ATE): green for no and red for yes; Previous surgery: blue for no and red for yes; Previous radiotherapy: blue for no and red for yes; Hemoglobin (Hb): on a color scale from 11 to 15 (light blue to dark blue); Leukocytes: on a color scale from 5 to 9 (light blue to dark blue); Platelets: on a color scale from 200 to 500 (light blue to dark blue); Cancer: cancer type, blue for head and neck cancer, red for non-small cell lung cancer (NSCLC); Cluster: hierarchical clustering using Ward’s minimum variance method with Euclidean distance.

Supplementary Figure 3. Mean differences in protein levels that decreased during chemotherapy or ICI compared with baseline**.** The horizontal dotted line indicates no difference. Dots and error bars represent mean differences with 95% confidence intervals. Results for chemotherapy are shown in light blue and for ICI in orange. The asterisk (*) next to the protein names indicates which proteins remain significantly decreased after FDR correction ICI: immune checkpoint inhibitors

Supplementary Figure 4. Mean differences in protein levels that increased during chemotherapy or ICI compared with baseline. The horizontal dotted line indicates no difference. Dots and error bars represent mean differences with 95% confidence intervals. Results for chemotherapy are shown in light blue and for ICI in orange. The asterisk (*) next to the protein names indicates which proteins remain significantly decreased after FDR correction. ICI: immune checkpoint inhibitors

Supplementary Figure 5. Mean differences in protein levels that decreased during chemotherapy in patients with NSCLC compared with baseline. The horizontal dotted line indicates no difference. Dots and error bars represent mean differences with 95% confidence intervals. Results for chemotherapy are shown in light blue and for ICI in orange. The asterisk (*) next to the protein names indicates which proteins remain significantly decreased after FDR correction ICI: immune checkpoint inhibitors, NSCLC: non-small cell lung cancer

Supplementary Figure 6. Mean differences in protein levels that increased during chemotherapy in patients with NSCLC compared with baseline. The horizontal dotted line indicates no difference. Dots and error bars represent mean differences with 95% confidence intervals. Results for chemotherapy are shown in light blue and for ICI in orange. The asterisk (*) next to the protein names indicates which proteins remain significantly decreased after FDR correction. ICI: immune checkpoint inhibitors; NSCLC: non-small cell lung cancer

Supplementary Figure 7. Mean differences in protein levels that decreased during chemotherapy in patients with head and neck cancer compared with baseline. The horizontal dotted line indicates no difference. Dots and error bars represent mean differences with 95% confidence intervals. Results for chemotherapy are shown in light blue and for ICI in orange. The asterisk (*) next to the protein names indicates which proteins remain significantly decreased after FDR correction ICI: immune checkpoint inhibitors

Supplementary Figure 8. Mean differences in protein levels that increased during chemotherapy in patients with head and neck cancer compared with baseline. The dotted line indicates no difference. Dots and error bars represent mean differences with 95% confidence intervals. Results for chemotherapy are shown in light blue and for ICI in orange. The asterisk (*) next to the protein names indicates which proteins remain significantly decreased after FDR correction ICI: immune checkpoint inhibitors

Supplementary Figure 9. Heatmap depicting mean changes in protein levels between baseline and three months, and their associations with clinical outcomes during follow-up. The patients are on rows, mean differences of proteins between 3 months and baseline in columns (positive [red] and negative [blue] values indicate increases and decreases in protein concentrations over time, respectively.), Unsupervised hierarchal clustering is shown on the top and left sides of the heatmap and on the zoomed dendrogram in panel B. Annotations on the right-side show: Thromboembolism (TE): arterial or venous thrombosis, blue for no and red for yes; Bleeding: major bleeding or clinically relevant non major bleeding, blue for no and red for yes; Mortality: blue for no and red for yes; Progression: treatment failure, blue for no and red for yes Therapy: light blue for chemotherapy and orange for ICI (immune checkpoint inhibitors).
